# Public funding for research on antibacterial resistance in the JPIAMR countries, the European Commission, and related European Union agencies: a systematic observational analysis

**DOI:** 10.1016/S1473-3099(15)00350-3

**Published:** 2016-04

**Authors:** Ruth Kelly, Ghada Zoubiane, Desmond Walsh, Rebecca Ward, Herman Goossens

**Affiliations:** a Medical Research Council, London, UK; b Department of Medical Microbiology, Vaccine & Infectious Disease Institute, University of Antwerp, Antwerp, Belgium

## Abstract

**Background:**

Antibacterial resistant infections are rising continuously, resulting in increased morbidity and mortality worldwide. With no new antibiotic classes entering the market and the possibility of returning to the pre-antibiotic era, the Joint Programming Initiative on Antimicrobial Resistance (JPIAMR) was established to address this problem. We aimed to quantify the scale and scope of publicly funded antibacterial resistance research across JPIAMR countries and at the European Union (EU) level to identify gaps and future opportunities.

**Methods:**

We did a systematic observational analysis examining antibacterial resistance research funding. Databases of funding organisations across 19 countries and at EU level were systematically searched for publicly funded antibacterial resistance research from Jan 1, 2007, to Dec 31, 2013. We categorised studies on the basis of the JPIAMR strategic research agenda's six priority topics (therapeutics, diagnostics, surveillance, transmission, environment, and interventions) and did an observational analysis. Only research funded by public funding bodies was collected and no private organisations were contacted for their investments. Projects in basic, applied, and clinical research, including epidemiological, public health, and veterinary research and trials were identified using keyword searches by organisations, and inclusion criteria were based on the JPIAMR strategic research agenda's six priority topics, using project titles and abstracts as filters.

**Findings:**

We identified 1243 antibacterial resistance research projects, with a total public investment of €1·3 billion across 19 countries and at EU level, including public investment in the Innovative Medicines Initiative. Of the total amount invested in antibacterial resistance research across the time period, €646·6 million (49·5%) was invested at the national level and €659·2 million (50·5%) at the EU level. When projects were classified under the six priority topics we found that 763 (63%) of 1208 projects funded at national level were within the area of therapeutics, versus 185 (15%) in transmission, 131 (11%) in diagnostics, 53 (4%) in interventions, and only 37 (3%) in environment and 39 (3%) in surveillance.

**Interpretation:**

This was the first systematic analysis of research funding of antibacterial resistance of this scale and scope, which relied on the availability and accuracy of data from organisations included. Large variation was seen between countries both in terms of number of projects and associated investment and across the six priority topics. To determine the future direction of JPIAMR countries a clear picture of the funding landscape across Europe and Canada is needed. Countries should work together to increase the effect of research funding by strengthening national and international coordination and collaborations, harmonising research activities, and collectively pooling resources to fund multidisciplinary projects. The JPIAMR have developed a publicly available database to document the antibacterial resistance research collected and can be used as a baseline to analyse funding from 2014 onwards.

**Funding:**

JPIAMR and the European Commission.

## Introduction

Over the past decade the emerging global threat of antibacterial resistance has alarmingly come to the forefront. Antibiotics, thought one of the greatest medical discoveries of the 20th century, and still pivotal in medicine now, are becoming increasingly compromised. The diminishing effectiveness of many antibiotics is due to the emergence of antibacterial resistance, and although a natural phenomenon, the inappropriate use of antibiotics in both human beings and animals worldwide, has accelerated the emergence and spread of highly resistant bacterial clones.^1^, ^2^ As regularly highlighted, between 1929 and the 1970s, 20 new classes of antibiotics were introduced to the market; since then, there has been a discovery void, with only two new classes reaching this stage.^3^ The speed at which bacteria have evolved to become resistant to antibiotics has surpassed the speed of drug discovery. This exacerbates the issue of resistance and stresses the need to preserve the efficacy of existing antibiotics. In short, without effective treatment, not only would bacterial epidemics become a substantial public health threat once again, but advances in modern medicine, ranging from minor surgery to cancer therapy, would also be jeopardised.^3^

**Research in context**

**Evidence before the study**

On Jan 5, 2015, we searched PubMed and Google using the following search terms: “antibiotic research funding”, “antibiotic resistance research funding”, “antimicrobial research funding”, “antimicrobial resistance research funding”, “antibacterial resistance research funding”, “antibacterial research funding”, “AMR funding”, “infection research funding”, “bacteriology research funding”, for manuscripts published between Jan 1, 1995, and Jan 5, 2015, with no language restrictions. Our search identified two studies with some relevance to this study. Many reports and papers exist detailing the threat of antibacterial resistance; however, what research had been funded to address this pressing public health issue within and between countries was unknown. We concluded that the scale and scope of the two similar studies identified investigating the UK (Head and colleagues, 2014, and Bragginton and Piddock, 2014) were limited, only taking the UK into account, and their inclusion criteria did not span the breadth of our work. The Joint Programme–Neurodegenerative Disease initiative did a similar mapping exercise in 2011, which we took into consideration when designing this study (Joint Programme–Neurodegenerative Disease Mapping Exercise Report, 2011). Since we did not identify any similar exercise in antibacterial resistance, we did a comprehensive analysis to identify all the research funded across 19 Joint Programming Initiative on Antimicrobial Resistance (JPIAMR) countries and European Union (EU)-level organisations (Directorate General for Research and Innovation [DG Research], European Centre for Disease Prevention and Control [ECDC], Directorate General for Health and Consumer Affairs [DG-SANCO], and Innovative Medicines Initiative) related to antibacterial resistance from 2007 to 2013 to have the evidence base to influence future work. The JPIAMR strategic research agenda provided details on the six priority topics into which the research projects were categorised to produce meaningful results.

**Added value of this study**

This study takes a widely holistic and encompassing approach to look at a range of research areas that the JPIAMR strategic research agenda has identified as priorities to tackle the global problem of antibacterial resistance. We included research into treatments and preventive measures in human and veterinary medicine, diagnostics, surveillance, transmission, and interventions to prevent resistance emergence, acquisition, transmission, and infection. Additionally, this study analysed data from 19 countries (17 European countries, Canada, and Israel) and the EU, including DG Research (Framework Programme 6 and 7, European Research Council, and Innovative Medicines Initiative), DG-SANCO, and the ECDC. Hence, this study was the first comprehensive systematic analysis of research specifically relevant to antibacterial resistance. By including veterinary, public health, infection control, and diagnostic research in our study, we are better able to define the scale and scope of research to reduce the burden of antibacterial resistance at a national level across 19 JPIAMR countries and at the EU level through the European Commission and related EU agencies. Results for the UK were compared with Head and colleagues, 2014, and Bragginton and Piddock, 2014, who also analysed UK data. However, their inclusion criteria did not span the breadth of this study (since we also included measures to prevent antibacterial resistance, optimisation of the use of existing antibiotics, diagnostics, surveillance, veterinary and environmental research, and interventions). Additionally, data for this study were mainly extracted through internal databases, probably resulting in more hits than manual searching through websites as was done for the two UK studies.

**Implications of all the available evidence**

Both UK-based studies and several other reports have claimed investment in antibacterial resistance research is inadequate when compared with the burden of resistance or investment in other areas of health research. We too came to the same conclusions; however, although the provision of new funds is necessary, what these studies did not address is that the burden of antibacterial resistance cannot be tackled by focusing solely on antibiotic development research. To overcome this global threat, a multidisciplinary and transnational approach is needed and all priorities identified by the JPIAMR strategic research agenda require due consideration and investment. Overall, our results provide a baseline from which JPIAMR countries and the EU-level organisations can measure their investment in this area from 2013 onwards and could provide the evidence needed to influence practice and policy at the national level and EU level. This comprehensive analysis also lays the groundwork for future follow-on studies in the area to capture the number of new initiatives in antibacterial resistance in recent times, such as the UK antimicrobial resistance cross-council initiative and the diagnostic prizes announced by the UK and the European Commission, among others. The database of funded research on antibacterial resistance being created and maintained by the JPIAMR will help facilitate studies on antibacterial resistance more readily in the future.

In Europe alone, an estimated 25 000 people died from resistant bacterial sepsis in 2007, costing €1·5 billion, placing a substantial strain on already stressed health budgets.^4^ A review^5^ in 2014 estimated that an additional 10 million lives a year will be lost by 2050 worldwide as a result of antimicrobial resistance in six key pathogens, four being bacterial, resulting in a cumulative cost of US$100 trillion. Because of the assumptions made and data analysed, these figures are predicted to be an underestimate,^5^, ^6^ but the trends are clear and cannot be ignored.

We are rapidly returning to a pre-antibiotic era, potentially resulting in the next world health crisis. The pressing urgency of this issue has been pointed out by several organisations worldwide, including WHO, with the poignant slogan used on World Health Day 2011, “No action today—no cure tomorrow”.^7^

Antibacterial resistance is a multifaceted problem needing vast and versatile solutions. No individual sector or nation has the capacity to independently handle this major societal challenge. Therefore, to collectively address antibacterial resistance at a national level and to increase the current impact of public research through more effective, efficient, and aligned investment, the Joint Programming Initiative on Antimicrobial Resistance (JPIAMR) was established in 2011.^8^ This initiative brings together 19 JPIAMR member countries, consisting of 17 European countries (Belgium, Czech Republic, Denmark, Finland, France, Germany, Greece, Italy, the Netherlands, Norway, Poland, Romania, Spain, Sweden, Switzerland, Turkey, the UK), Canada, and Israel, with Estonia and Argentina as JPIAMR observers.

The JPIAMR launched its strategic research agenda in April, 2014, outlining the member states' common vision to tackle antibacterial resistance.^8^ To ensure comprehensive actions are pursued, the strategic research agenda identifies six holistic and encompassing priority topics. The strategic research agenda acts as a dynamic framework on which the JPIAMR will continue to launch joint activities to guide and align research and investment to reduce the burden of antibacterial resistance across Europe and beyond. The JPIAMR will maintain and extend engagement activities internationally with different stakeholders, including industry, health service organisations, policy makers, the European Commission, and the research community, among others.

To guide future research activities and underpin the implementation of the JPIAMR strategic research agenda, an understanding of the present research landscape is necessary, which can be achieved by obtaining an objective insight into the scale and scope of research specifically relevant to antibacterial resistance across JPIAMR countries, the European Commission, and related European Union (EU) agencies. No detailed analyses on research portfolios and associated investment to address antibacterial resistance—including human, veterinary, and environmental research—across multiple countries have been done previously. In this Article we present in-depth analyses examining research specifically relevant to antibacterial resistance from major funding organisations funded within a 7 year period (2007–13) across the 19 JPIAMR countries and at EU level. The EU-level funding includes funding from the Directorate General for Research and Innovation (DG Research) through Framework Programme (FP) 6 and FP7 (including the Innovative Medicines Initiative first programme [IMI-1] and the European Research Council [ERC]), the Directorate General for Health and Consumer Affairs (DG-SANCO), and the European Centre for Disease Prevention and Control (ECDC). We will then provide recommendations for how JPIAMR and member countries could proceed in the future.

## Methods

### Participating countries and data sources

We did a systematic observational analysis surveying the 19 JPIAMR countries to establish levels of publicly funded research into antibacterial resistance. A questionnaire was sent to, and completed by, all JPIAMR national representatives who contacted suitable public funding agencies within their respective country. The full list of funding agencies included in this survey and the questionnaire used are in the appendix. All national representatives were briefed on the survey through presentations and discussions at JPIAMR meetings and through one-to-one communications with the data analyser. The variables collected included organisation name, principal investigator, lead institution, title, abstract or summary, start and end dates, and the total investment in euros. Only research funded by public funding bodies (which can invest in both public and private organisations) was collected and no private organisations were contacted for their investments because of difficulties in obtaining data. Data were also provided by DG Research (including FP6, FP7, ERC, and IMI), DG-SANCO, and ECDC, hereby collectively referred to as EU level. Additional desk study was done, mainly using websites and published databases from research organisations, at national and EU level, to identify through keyword searches additional publicly funded research that might have been missed. These projects were then verified for inclusion by the organisation or national representative. The data collected were the most comprehensive data available at the time of the survey from the organisations contacted.

We used the title and abstract where available (an abstract or summary was available for 984 [87%] of the 1129 JPIAMR country-level and 112 [98%] of the 114 EU-level projects) as a filter to establish research specifically relevant to antibacterial resistance. The inclusion criteria were based on the six encompassing priority topics identified in the JPIAMR strategic research agenda. This Article concentrates on all active and completed research projects with committed funding of €100 000 or more from January, 2007, up to, and including, December, 2013, in basic, applied, and clinical research, including trials, epidemiological, public health, and veterinary research. Some projects might not have begun spending until early 2014 and projects less than €100 000 were not regarded by funders as research projects but as network funding and therefore were not captured for this Article. Projects included were in the areas of therapeutics, such as from basic research to market, ranging from understanding the molecular mechanisms of resistance, to the development of new antibiotics and therapeutic alternatives to antibiotics (such as antivirulence drugs, vaccines, coatings on implants, bacteriophages, drug delivery, etc), and the optimisation of the use of existing antibiotics (eg, stewardship and clinical trials on combination treatment options); development of new diagnostics, such as a point-of-care test to effectively differentiate between bacterial and viral infections, or a rapid test to identify resistant bacteria and its resistance or sensitivity profile; surveillance, such as monitoring resistance rates, or antibiotic use in human or agricultural settings at local, national, or international levels; the transmission dynamics of resistance between different (human and animal) reservoirs; the assessment of the effect of environmental pollution (eg, water, soil, sewage) containing antibiotics, antibiotic residues, and resistant bacteria on the spread of resistance; and interventions to prevent the acquisition, transmission, and infection caused by antibacterial resistant bacteria (eg, infection control procedures, hospital layout, and education programmes).

Since funding mechanisms across different countries and agencies vary and to maximise consistency, investment allocated to infrastructure (eg, buildings, faculties, and networks) was not included, unless embedded within large grants.

All financial information is as reported by the funders; grants awarded in a currency other than euros were converted to euros by the organisations at the time of data collection. No adjustments were made by the data analyst for inflation since information about whether adjustments for inflation had been previously made by individual organisations was not available before submission.

### Statistical analysis

To ensure the data collected met the established inclusion criteria, that no duplication of projects occurred, and that the data provided were complete and accurate, all data were checked and validated twice. First, this was done by the representatives within each participating JPIAMR country and EU-level organisations, and second, in more detail, by the data analyser to ensure consistency across funding organisations. For projects where only a proportion of the project met the inclusion criteria, for example a project looking at fungal and bacterial resistance, only a proportion of funding was allocated to this project; this was done on a case-by-case basis by the data analyser. To ensure consistent classification of projects, the data analyser read the title, abstract, and any further information of each individual project and classified them into one or more of the six priority topics. The JPIAMR Scientific Advisory Board was consulted for any uncertainties.

Data were sourced, categorised, and analysed during the period of July 1, 2013, to Feb 1, 2015. Data analyses and generation of figures and graphs were done with Microsoft Excel 2010.

### Role of the funding source

The funders of the study had no role in the study design, data analysis, data interpretation, or writing of the Article. Members of the JPIAMR and the European Commission provided data included in this work. To the authors' knowledge, all data analysed for this Article are already publicly available and therefore have no restrictions. The corresponding author had full access to all the data in the study and had final responsibility for the decision to submit for publication.

## Results

We identified 1234 publicly funded antibacterial resistance projects across 19 JPIAMR countries and at the EU level, with a total investment of €960·7 million. Additionally, DG Research invested in another nine antibacterial resistance research projects via the IMI-1 programme in partnership with the European Federation of Pharmaceutical Industries and Associations, bringing the total public investment in antibacterial resistance research from 2007 to 2013 across 1243 projects to more than €1·3 billion (table). We found the 19 JPIAMR countries collectively contributed €646·6 million (67%) of the €960·7 million total investment in antibacterial resistance research, whereas the remaining €314·1 million (33%) was provided at the EU level. However, when DG Research's contribution to the IMI-1 is considered, JPIAMR countries accounted for only 49·5% (€646·6 million of €1·3 billion) of this investment and 50·5% (€659·2 million of €1·3 billion) was provided at the EU level (table). We investigated patterns over time by analysing the committed budget per year in 19 JPIAMR countries collectively and in EU-level organisations, where positive patterns in funding from 2007 to 2013 were observed (appendix).

At the national level, we identified 1129 projects funded across 19 JPIAMR countries, with a total public sector investment of €646·6 million across the 7 year period (2007–13). Most public sector funding went to universities, some went to hospitals, and only a small proportion went to private organisations.

We analysed the total number of projects and associated investment within each of the strategic research agenda priority topics and between countries. Because antibacterial resistance is a complex issue, some multidisciplinary projects spanned more than one priority topic. As such, 69 (6·1%) of the 1129 projects in total funded at national level are classified under more than one priority topic, slightly inflating the total number of projects to 1208. Overlap was most evident between therapeutics and transmission in basic underpinning science projects and between transmission, environment, and surveillance. No duplication of funding occurred since an equal proportion of investment was assigned to each priority topic in projects classified under more than one priority topic.

**Figure 1:**
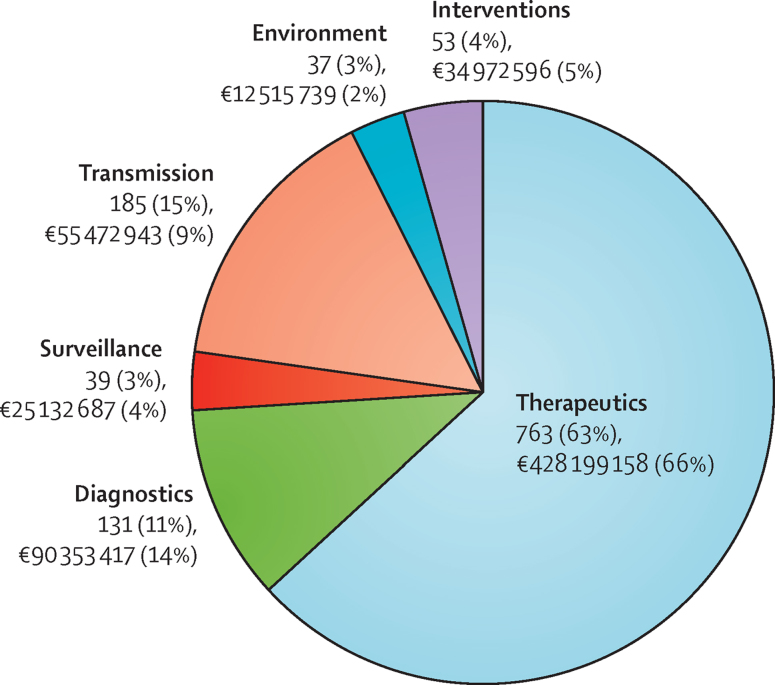
Antibacterial resistance projects funded at national level between 2007 and 2013 by priority topic with total funding Proportions of pie chart represent number of projects by priority topic and not total funding. Projects funded at the European Union level are not included. Some projects are classified under more than one priority topic; hence, the numbers of projects are duplicated. Investment is not duplicated because a percentage of investment has been assigned to each priority topic in projects classified under more than one priority topic. Percentages do not add up to 100% because of rounding.

Of 1208 projects investigated, with €646·6 million invested overall, 763 (63%) were within the area of therapeutics, with a total investment of €428·2 million (66%); 185 (15%) were based on the transmission of antibacterial resistance, with a total investment of €55·5 million (9%); 131 (11%) were about the development of new diagnostics, with a total investment of €90·4 million (14%); 53 (4%) were within the area of interventions, with a total investment of €35 million (5%); 39 (3%) were within the area of surveillance, with a total investment of €25·1 million (4%); and 37 (3%) focused on the environment, with a total investment of €12·5 million (2%; figure 1).

**Figure 2:**
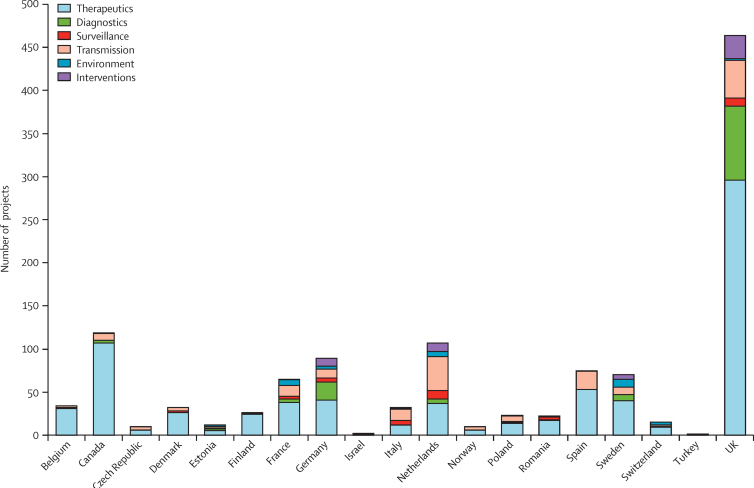
Total number of projects per country by priority topic funded at national level Totals include national data from participating countries from 2007 to 2013 and do not include projects funded at the European Union level within these countries. Some projects are classified under more than one priority topic (hence, the numbers of projects are duplicated).

**Figure 3:**
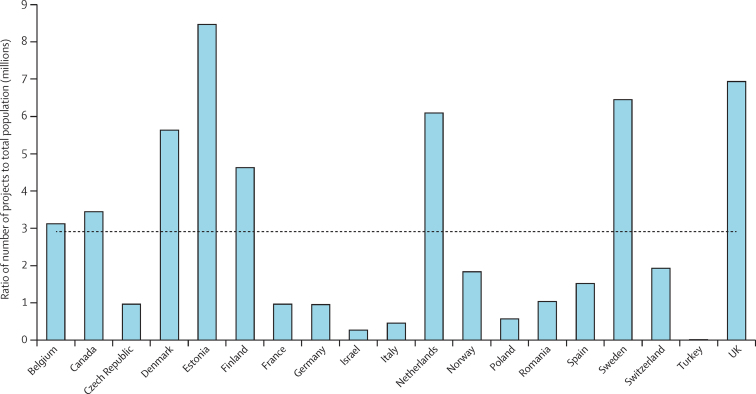
Ratio of total number of projects per country to the total national population in millions Total number of project includes national data from participating countries from 2007 to 2013 and does not include projects funded at European Union level. The mean country population from 2007 to 2013 was used.^9^, ^10^ The mean ratio is shown by the dotted line.

We identified substantial variations in funding across countries at the national level, both in terms of number of projects and investment. Figure 2 shows the number of projects per country by priority topic; similar results were evident for investment (appendix). In an attempt to gauge the number of projects in this area per person of population, we also analysed the ratio of the number of projects to the population of that country (figure 3). The mean country population from 2007 to 2013 was used.^9^, ^10^ Again, substantial variation existed between countries in the number of projects per person of the population with a broad spread around the mean, but the data suggested that Denmark, Estonia, Finland, the Netherlands, Sweden, and the UK funded more projects than the other countries included (figure 3). Similar results were evident for the ratio of investment per person of the population (appendix).

We found that EU-level organisations invested €314·1 million in 105 research projects specifically related to antibacterial resistance from 2007 to 2013 via DG Research (FP6, FP7, ERC), DG-SANCO, and ECDC (DG Research's contribution to IMI is not shown). Since substantial investment was made through large multinational consortia, 19 (18%) of the 105 projects were classified under more than one priority topic. No duplication of funding occurred since a percentage of investment has been assigned to each priority topic in projects classified under more than one priority topic.

**Figure 4:**
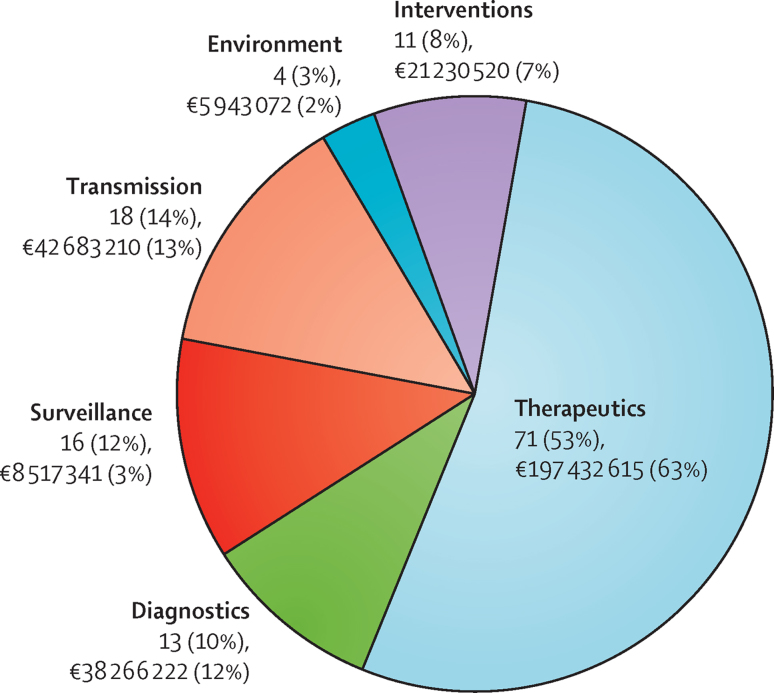
Antibacterial resistance projects funded at the European Union level between 2007 and 2013 by priority topic with total funding Proportions of pie chart represent number of projects by priority topic and not total funding. European Union level funding collectively refers to funding by the Director General for Research and Innovation (Framework Programme 6 and 7 and the European Research Council), the Directorate General for Health and Consumer Affairs, and the European Centre for Disease Prevention and Control; Director General for Research and Innovation's contribution to Innovative Medicines Initiative first programme is not included in this chart. Some projects are classified under more than one priority topic; as such, the numbers of projects are duplicated. Investment is not duplicated within the priority topics because a percentage of investment has been assigned to each priority topic in projects classified under more than one priority topic.

Of the 133 projects funded at the EU level (105 projects plus the duplicates), receiving an investment of €314·1 million overall, 71 (53%) were in the area of therapeutics, with a total investment of €197·4 million (63%); 18 (14%) were classified as transmission and received an investment of €42·7 million (13%); 16 (12%) were classified as surveillance, but only received an investment of €8·5 million (3%); 13 (10%) were classified as diagnostics and received €38·3 million (12%); 11 (8%) were classified as interventions, with investments of €21·2 million (7%); and four (3%) were classified as the environment, with investments of €5·9 million (2%; figure 4).

We captured nine projects within IMI-1 meeting the study inclusion criteria, with a total investment of €723·5 million. DG Research has committed €345·1 million to these nine projects via FP7. Most projects address more than one JPIAMR priority topic, although funding is mainly within priority topic A: therapeutics, to strengthen clinical research on antibacterial resistance in Europe. In addition to preclinical and clinical research, the IMI-1 funded projects also focus on developing research infrastructure—for example, all projects, with the exception of one, are required to submit information to a specific database, and three projects aim to develop a drug discovery platform and pan-European clinical trials and laboratory networks.

## Discussion

To our knowledge, this study is the first systematic analysis of research funding specifically relevant to antibacterial resistance across 19 countries, and at EU level, including the IMI-1. This study was also the first, to our knowledge, to take human, veterinary, and environmental research, and all areas identified in the JPIAMR strategic research agenda, including therapeutics, diagnostics, surveillance, transmission, environment, and interventions, into account. IMI-1, the world's largest public–private partnership in life sciences, involving the European Commission and European Federation of Pharmaceutical Industries and Associations, was important to capture as the programme is investing substantially to accelerate the development of better and safer medicines for patients, including antibiotics and alternative treatments for bacterial infections.^11^

When looking at the total spend and number of awards made in antibacterial resistance research from 2007 to 2013, investment across the JPIAMR countries seems to be substantial. However, when compared with total spend on research across the disciplines, the amount spent on antibacterial resistance seems to be very small. For example, in the UK, one of the biggest investors, antibacterial resistance research spend was about 1% of the total research spend for the same period for the four research councils included.^12^, ^13^, ^14^, ^15^, ^16^, ^17^, ^18^, ^19^, ^20^, ^21^, ^22^ Also evident from the analyses of available data is that, at the EU level, substantially more was invested in antibacterial resistance research in 2007–13 (€314 million and an additional €345 million in IMI-1) than by the JPIAMR countries, which collectively invested €647 million. Therefore, not taking into account the DG Research's contribution to the IMI-1, 33% of the total investment was at the EU level versus 67% from all 19 countries (table). By contrast, when looking at other major societal challenges, such as neurodegenerative diseases, the proportion of annual funding allocated in projects active on Jan 1, 2011, by DG Research (FP7) was 15% (€57 million) versus 85% (€314 million) by the 20 countries participating in the Joint Programme for Neurodegenerative Disease.^23^ This proportion of funding invested in neurodegenerative disease research by DG Research is more in line with the EU research FP spend. As in 2007–08, EU FP funds represented about 7·5% of all civil research and development expenditure financed by governments of EU member states and European Free Trade Association countries. Although we recognise the 7·5% captures all civil research and development spend, comparing it with what we found (33% funded at the EU level *vs* 67% by governments in antibacterial resistance research; table) emphasises the need for national budgets to redress the balance.^24^

Furthermore, countries varied substantially in terms of total number of antibacterial resistance projects funded (figure 2) and number of projects funded per person of population (figure 3), with Denmark, Estonia, Finland, the Netherland, Sweden, and the UK investing in a greater number of projects than the other countries investigated. Interestingly, those countries that invest the most have lower levels of antibacterial resistance than many of the countries included in this Article that invest the least.^25^ Also, within countries, funding for other high priority health needs varied substantially—for example, according to the Joint Programme for Neurodegenerative Disease findings, Germany was the biggest funder of neurodegenerative disease research,^23^ yet, we found Germany was only a very minor funder of antibacterial resistance research. This disparity between funding stresses the need for new funds for antibacterial resistance within countries rather than redistributing funds from other essential areas of research. The exact reasons for the disparate funding within and between countries are unclear, but we believe they argue for improved coordination between countries to share best practice and experience and to embark on joint research projects. We also believe these observations argue for greater sharing of results and data so that the outputs and effects of large investments can be realised across the EU and beyond.

The burden of disease caused by antibacterial resistance is on the increase. In 2007, 25 000 people died in Europe from resistant bacterial sepsis, costing €1·5 billion.^4^ The 2014 review from the O'Neill commission^5^ established by the UK Government estimated that an additional 10 million lives per year will be lost by 2050 globally as a result of antimicrobial resistance in six key pathogens, resulting in a cumulative cost of US$100 trillion. This impending health challenge clearly needs to be addressed through research, but this research also needs to extend beyond the boundaries of individual states. The number of projects funded by individual states is high; 1129 (91%) of the 1243 projects identified in this survey were funded at the national level, but they only account for €646·6 million (49%) of the total investment, suggesting that these are relatively small awards and highly focused. These awards would certainly have been made on the basis of scientific excellence through normal schemes.

In view of the relatively small amount of funds invested at the national level in antibacterial resistance compared with the EU level, other health priorities, and other countries, changes might now be needed at the national level in countries that correspond with the strategic importance and growth of antibacterial resistance. The idea behind joint programming is that resources are scarce and the availability of public investments in research has limits.^26^ Hence, participating countries will not only need to close the gap between the health research needs and the actual research funded but also make strategic and coordinated investments with existing and new funds. These strategies entail that countries work both alone and together in a more efficient way than they are now to increase the effectiveness of research through strengthening national and international coordination and collaborations, harmonising research activities, and collectively pooling resources. From our analysis, some countries already invest substantial funds into antibacterial resistance but a new way of funding multidisciplinary and transnational antibacterial resistance research is needed. The UK Cross Research Council Initiative aims to achieve this goal, not only by linking different research disciplines, both nationally and internationally, but also through coordination of different sectors and funders through the UK antimicrobial resistance funders forum.^27^, ^28^

The JPIAMR has also developed the strategic research agenda to coordinate research in close collaboration with the funding instruments of the European Commission, specifically Horizon 2020, IMI, and the ERA-NET scheme to increase effectiveness and avoid duplication. Countries could use the JPIAMR strategic research agenda now as a template for coordination. Beyond Europe, government priorities are beginning to change, and new increased budget commitments are being discussed, with the US Government proposing to invest more than $1·2 billion of funding for 2016 to improve antibiotic stewardship, strengthen antibiotic resistance risk assessment, surveillance, and reporting capabilities, and drive research and innovation in the human health and agricultural sectors.^29^ Activity within the area of diagnostics has also increased, with the US Department of Health and Human Services announcing a diagnostic prize of up to $20 million,^30^ the European Commission announcing a €1 million diagnostic prize,^31^ and the UK announcing a £10 million Longitude prize in diagnostics.^32^

As with similar exercises,^33^, ^34^ the data presented have limitations. To ensure the information captured was comparable and consistent, only research projects were captured, and institutional funding was unobtainable. Additionally, because the remit of the study was to capture research projects only, surveillance systems that are now core programmes—such as ECDC in-house programmes and national surveillance programmes, including the German KISS and the French RAISIN programmes, among others—were not captured in this study and therefore the picture of surveillance funding captured by this study is not comprehensive. We relied on the accuracy of the data provided by funding bodies, although all data were verified by each national or EU representative and any apparent discrepancies or duplication of projects were dealt with by the data analyst.

All projects were determined for inclusion, first, by each organisations representative, and second, by the data analyst. Some projects might have been missed because of the search terms used or the difficulties in accessing data in some countries. The rules regarding what a research grant will cover can vary between countries (eg, salary of investigators); we made no attempt to remove any indirect and estate costs included in the funding amounts and did not adjust for inflation. However, to deal with these financial confounders, we analysed the number of projects and compared trends across countries using number of projects rather than focusing solely on investment. If this exercise were to be extended globally and include countries such as China and India with fluctuating currency and inflation rates over time, the method described by Young and colleagues^35^ could be followed to ensure careful comparison between countries with variable exchange and inflation rates. The subjective classification of these cross-disciplinary projects, by the data analyst, to one or more of the priority topics leaves the exercise somewhat open to question. Additionally, for projects classified under more than one priority topic, we could not ascertain the exact proportion of grant funding to be allocated to each of the topics, hence, to ensure consistency an equal proportion of funding was assigned to each. Overlap was often evident between therapeutics and transmission in basic underpinning science projects and also between transmission, environment, and surveillance.

Currency conversions to euros will not be precise because of variations in the exchange rates across the data collection process. Although private funding invested in IMI-1 was available, this study did not include private sector funding because of difficulties in openly accessing private funding and project information beyond IMI-1.

As a result of this study, several recommendations have been made to the JPIAMR members to consider, with some activities already underway. At present, no comprehensive database exists to document research at both national and international levels, and, in view of this study, improvements in data sharing and communication clearly need to be achieved at the national level in several countries. The JPIAMR is actively working to improve data sharing and has turned the research data collected for this study into a useful, freely accessible, and searchable database available on the JPIAMR website. The database will enable researchers and funders to set strategic priorities by revealing what has already been funded across the different areas and what is still needed within the different priority areas. Furthermore, the database is intended to be used by researchers for networking and collaboration and to avoid duplication. If funders from other countries provide similarly detailed information about projects on antibacterial resistance research, global gaps and priorities could be assessed.

The substantial allocation of antibacterial resistance projects and investment within the area of therapeutics, both at the national and EU level, is probably because of the existing strength of basic bacteriology research across many of the JPIAMR countries. What will be important from now on is for the JPIAMR and other organisations to ensure that this level of basic research underpins the development of therapies, diagnostics, and intervention strategies for use in clinical and veterinary practice and in the environment to reduce resistance. However, the development of new treatment in a timely manner is challenging since antibiotics take years to develop; therefore actions need to be taken that can have an immediate effect on the rate of acquisition, transmission, and infection by resistant bacteria. Consequently, the JPIAMR have plans to launch a call in January, 2016, to boost funding in this key area, focusing on the One Health Agenda, which uses the ERA-NET scheme. Additionally, increased research effort in affordable, reliable, and rapid point-of-care diagnostics and interventions is necessary. Additionally, the effect of diligent hygiene practices and antimicrobial stewardship in human and agricultural settings, along with improved public and professional education, should not be underestimated.

To conclude, investment at present might not correspond with the burden of antibacterial resistance and the looming health, social, and economic threat it poses on the treatment of infections and on medicine in general. Antibacterial resistance clearly warrants increased and new investment from a range of sources, but improved coordination and collaboration with more informed resource allocation are needed to make a true impact. Hopefully, this analysis will prompt nations to pay due consideration to the existing research landscape when considering future investments. The analysis will act as a guide for the JPIAMR to ensure research is complementary and that no major overlaps exist, while aiming to identify gaps and opportunities to be exploited. The benefits of working across national boundaries, sharing experiences, and pooling resources are substantial, and these are yet to be fully taken advantage of by the JPIAMR countries. The entire JPIAMR strategic research agenda cannot be tackled at once by each country; thus, prioritisation of research within each country to where it is best placed to deliver useful results will ensure an efficient use of resources and avoid the duplication of efforts.

## Figures and Tables

**Table:** Total committed public funding to antibacterial resistance research by JPIAMR countries and the EU

	**Total number of projects, 2007–13**	**Total funding (€), 2007–13**	**Proportion of total funding (excluding EC contribution to IMI)**	**Proportion of total funding**
19 JPIAMR countries	1129	646 646 541	67·3%	49·5%
EU level*	114	659 201 418	NA	50·5%
EU level (excluding IMI)	105	314 072 980	32·7%	24·1%
IMI (EC contribution only)	9	345 128 438	NA	26·4%
Overall†	1243	1 305 847 959	100%	100%

The EU-level funding includes funding from the Director General for Research and Innovation (Framework Programme 6 and 7, including the IMI first programme and the European Research Council), the Directorate General for Health and Consumer Affairs, and the European Centre for Disease Prevention and Control from 2007 to 2013. JPIAMR=Joint Programming Initiative on Antimicrobial Resistance. EU=European Union. EC=European Commission. IMI=Innovative Medicines Initiative. NA=not applicable.

* Sum total of EU level (excluding IMI) and IMI (EC contribution only) funding.

† Sum total of 19 JPIAMR countries and EU level funding.
