# Supplementary material for: Public funding for research on antibacterial resistance in the JPIAMR countries, the European Commission, and related European Union agencies: a systematic observational analysis
Source: Lancet Infect Dis. 2016 Apr;16(4):431–40. doi: 10.1016/S1473-3099(15)00350-3 (PMC4802226; doi:10.1016/S1473-3099(15)00350-3)
Supplement: Supplementary appendix [file mmc1.pdf]

THE LANCET Infectious Diseases

Supplementary webappendix

This webappendix formed part of the original submission and has been peer reviewed. We post it as supplied by the authors.

Supplement to: Kelly R, Zoubiane Z, Walsh D, Ward R, Goossens H. Public funding for research on antibacterial resistance in the JPIAMR countries, the European Commission, and related European Union agencies: a systematic observational analysis. *Lancet Infect Dis* 2015; published online Dec 18. http://dx.doi.org/10.1016/S1473-3099(15)00350-3.

Supplementary Data 1: Funding bodies included in this work

Country	Public Funding Organisation
Belgium	Agency for Innovation by Science and Technology (IWT), Research Foundation Flanders (FWO), and Fonds de la Recherche Scientifique (FNRS)
Canada	Canadian Institutes of Health Research (CIHR), Natural Sciences and Engineering Research Council (NSERC). Other funding agencies were contacted but had no studies meeting the inclusion criteria.
Czech Republic	Internal Grant Agency of Ministry of Health (IGA) and Czech Science Foundation (GAČR)
Denmark	The Danish Council for Strategic Research and the Danish Council for Independent Research
DG SANCO	Directorate General for Health and Consumer Affairs
DG Research	Directorate General for Research and Innovation
ECDC	European Centre for Disease Prevention and Control
ERC	European Research Council
Estonia	Archimedes Foundation, Enterprise Estonia, Estonian Research Council, Ministry of Agriculture, Ministry of Education & Research
Finland	Academy of Finland
France	National Agency for Research (ANR), National Institute for Agricultural Research (INRA), Agency for Food, Environment and Occupational Health and Safety (ANSES), National Institute of Health and Medical Research (INSERM), the Medical Research Foundation (FRM), Hospital Clinical Research Program (PHRC), the Public Hospitals of Paris (AP-HP), the Ministry of Higher Education and Research, Ministry of Health, Single Inter-ministerial Fund, National Institution of Agriculture and Sea Products, the French Alternative Energies and Atomic Energy Commission, Carnot Institute for Animal Health (ICSA), Principality of Monaco, Association Raoul Follereau, and the Infectiopole-sud Foundation.
Germany	Projektträger Jülich (PtJ), Projektträger DLR (PT-DLR), and Deutsche Forschungsgemeinschaft (DFG)
IMI	Innovative Medicines Initiative (IMI) (EFPIA & EC- FP 7)
Israel	Ministry of Health
Italy	Chief Scientists Office, Ministry of Health (CSO-MOH)
The Netherlands	The Netherlands Organisation for Health Research and Development (ZonMw), plus additional desk study to identify publically available funded projects by other organisations
Norway	The Research Council of Norway (RCN)
Poland	National Science Centre (NCN) and National Centre for Research and Development (NCBR)
Romania	Ministry of National Education, the National Authority for Research, Technological Development and Innovation
Spain	Instituto de Salud Carlos III, Ministry of Economy and Competitiveness (MINECO), and Spanish Network for Research in Infectious Diseases (REIPI)
Sweden	Swedish Research Council (SRC), Vinnova, Forte, Formas, and the Swedish Foundation for Strategic Research (SSF)
Switzerland	Swiss National Science Foundation
Turkey	The Scientific and Technological Research Council of Turkey (TUBITAK)
The United Kingdom	Public funding bodies:<br>Department of Health (DH)/ National Institute of Health Research (NIHR), Medical Research Council (MRC), Biotechnology and Biological Sciences Research Council (BBSRC), Engineering and Physical Sciences Research Council (EPSRC), Natural Environment Research Council (NERC), Innovate UK, Department for Environment, Food and Rural Affairs (DEFRA), and the Food Standard Agency (FSA).<br>Charity:<br>The Wellcome Trust<br><br>The Economic and Social Research Council (ESRC), the Science and Technology Facilities Council (STFC), and the Arts and Humanities Research Council (AHRC) were contacted but did not have any projects meeting the study inclusion criteria.

Supplementary Data 2:

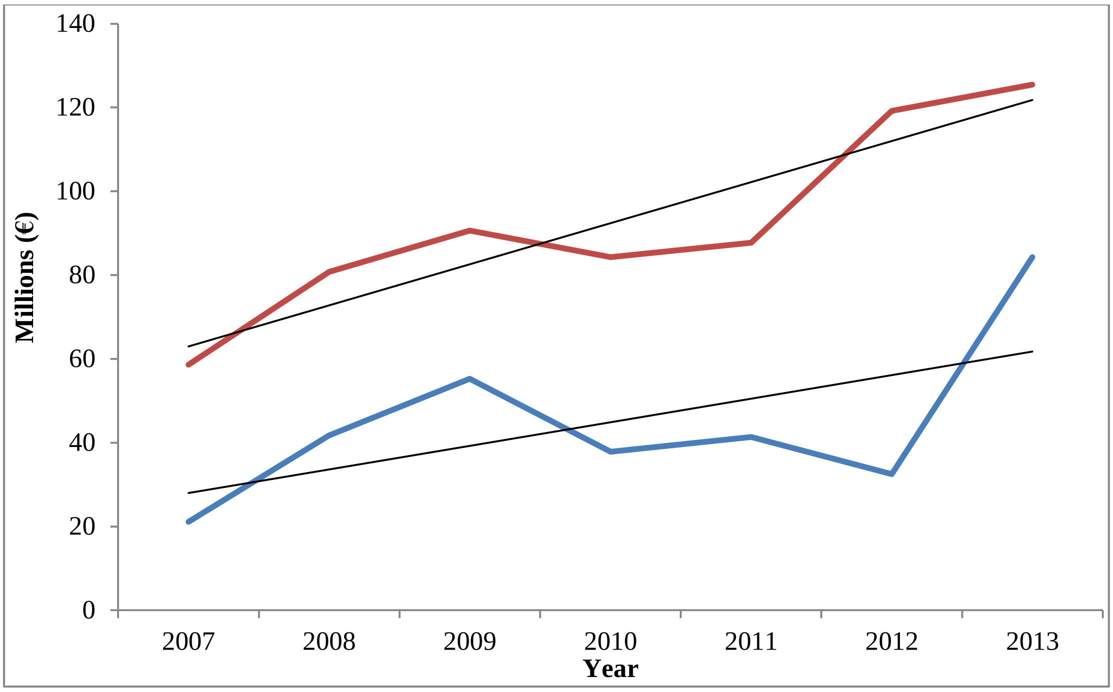

Committed budget per year by JPIAMR countries and at the EU-Level in antibacterial resistance research

The 19 JPIAMR countries are shown by the red line and EU-level data is shown by the blue line. EU-level data does not include DG Research's contribution to IMI-1.

Supplementary Data 3:

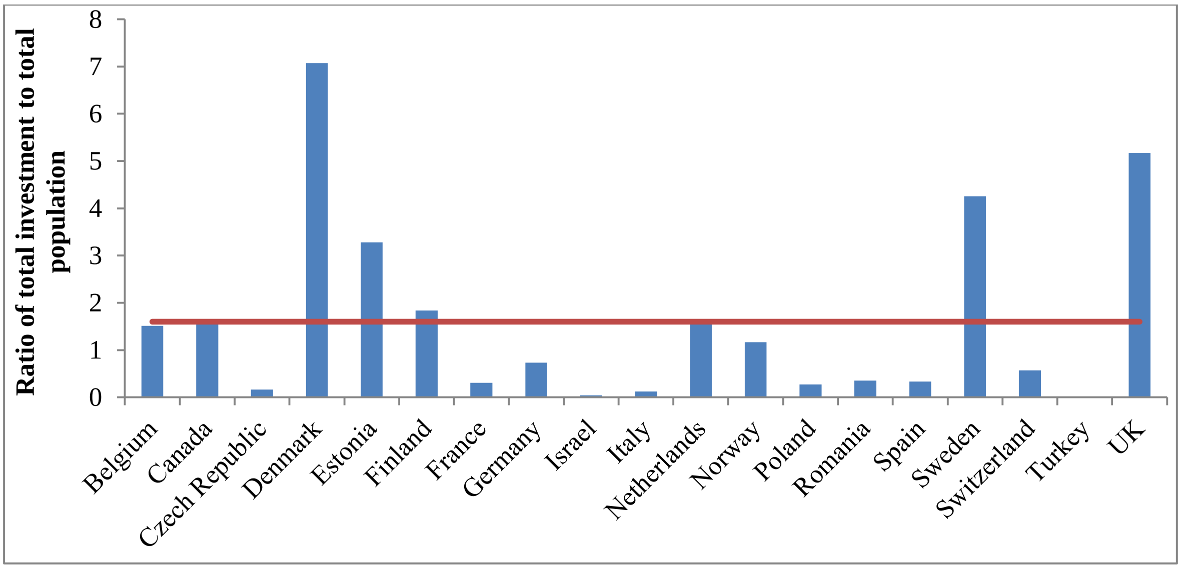

Ratio of total funding amount invested in antibacterial resistance research per country to the total national population of that country in millions

Total funding amount includes national data from participating countries from 2007 to 2013 and do not include projects funded at European Union-level. The mean country population from 2007 to 2013 was used. The mean ratio is shown by the red line.

Supplementary Data 4:

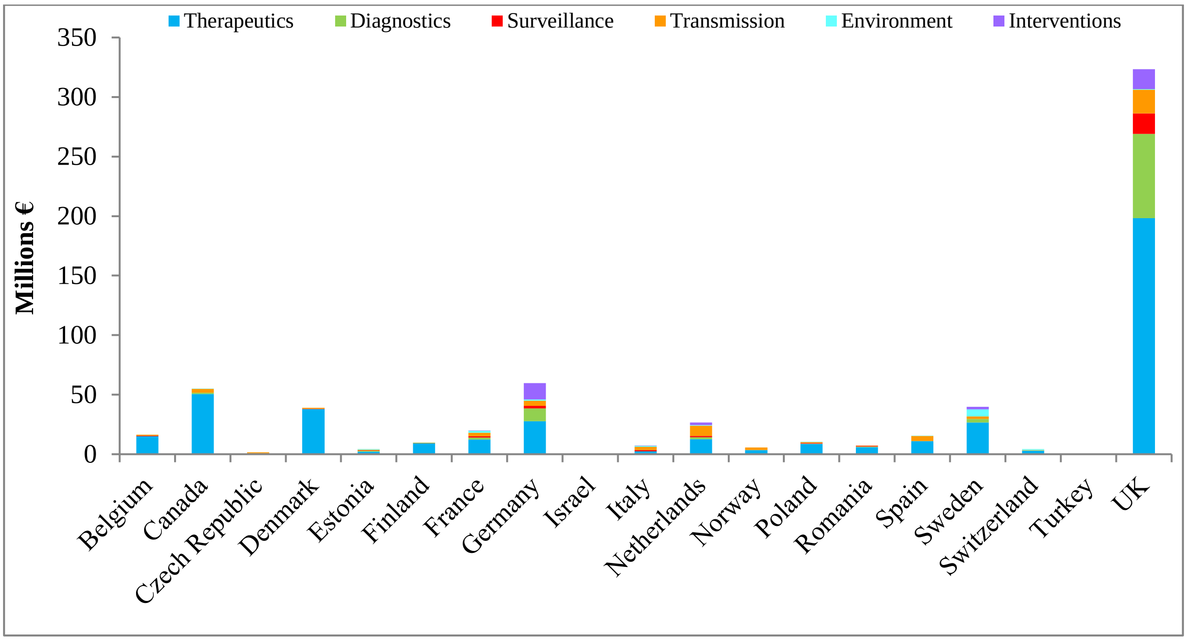

Total funding invested per country in antibacterial resistance research by priority topic funded at national level

Totals include national data from participating countries from 2007 to 2013 and do not include projects funded at European Union level within these countries.

Supplementary Data 5:

Questionnaire: Mapping Research in AMR across the Joint Programming Countries

The purpose of this questionnaire is to collate comprehensive information on funded research in the area of AMR (antibacterial resistance) in humans, animals and the environment across the JPIAMR countries.

The JPIAMR Strategic Research Agenda (SRA) and this mapping exercise will be used to help prioritise areas for implementation across the Joint Programming Initiative.

Guidance

Include:

- Please collate data from all relevant public funding organisations in your country (research councils, the department of health, environment, rural affairs, etc., charities….)

- Please list any grants ≥€100,000 funded in the area of AMR (based within the remit of the 6 Priority Topics of the SRA) since Jan 1, 2007 until Dec 31, 2013 [include both on-going and closed projects if funded from the beginning 2007].

- Please include all research grant funding covering basic, applied and clinical research, including trials and epidemiological studies, public health and veterinary research relevant to AMR

- Only research related to bacterial resistance should be included, not research on viruses, parasites, fungi, etc.

Exclude:

- Please exclude projects on basic bacteriology with no reference to bacterial resistance, antibiotic resistance, antibiotics, antibacterials in the title or abstract

- Projects on generic (e.g. buildings) and research infrastructure (e.g. facilities, networks)

If there is anything you are unsure of, please submit the project for further investigation.

If information is not available for a given question, please state 'NA' (not available) and please explain why this is the case.

Priority topics identified in the draft Strategic Research Agenda are:

A	Development of novel antibiotics and alternatives for antibiotics (drugs, biological, vaccines) – from basic research to the market
B	Design strategies to improve treatment and prevention of infections by developing new diagnostics
C	Global antibiotic resistance surveillance
D	Selection and transmission dynamics (biology and epidemiology)
E	The role of the environment and sewage as a source for the emergence and spread of antimicrobial resistance
F	Designing and testing Interventions to prevent acquisition, transmission and infection caused by antibiotic-resistant bacteria

If you have any questions, please contact the data analyst (details provided elsewhere).

Information requested:

Name	
Country	
Email address	
Funding Organisation	
Other funding organisations contacted (please list these here)	

Information requested for each project in excel format:

Funding organisation	Principal Investigator	Host institution	Title of grant	Summary/ Abstract	Funding (€)	Start (mm-yy)	End (mm-yy)
